# From Skeptic to Believer: Monitoring a Ring-Opening Isomerization of a CeIV Complex in the Solid-State Using a Home-Source Diffractometer

**DOI:** 10.1063/4.0000883

**Published:** 2025-10-27

**Authors:** Michael Gau

**Affiliations:** University of Pennsylvania, Philadelphia, PA, USA

## Abstract

As a service crystallographer, we hear all types of explanations as to why a crystal isn't what the intended researcher is looking for. A chemical transformation occurring in crystallo with no crystal decomposition was one I was always skeptical of. And here we are, 3 years into the journey of studying an in crystallo ring-opening of a CeIV complex. A series of metal-cyclopropenyl complexes will be presented and the focus will be on the trials and tribulations to discovering and closely analyzing the cerium congener that performs an in crystallo ring-opening.

